# Small Aperture IC-8 Extended-Depth-of-Focus Intraocular Lens in Cataract Surgery: A Systematic Review

**DOI:** 10.3390/jcm11164654

**Published:** 2022-08-09

**Authors:** José-María Sánchez-González, María Carmen Sánchez-González, Concepción De-Hita-Cantalejo, Antonio Ballesteros-Sánchez

**Affiliations:** 1 Vision Research Group (CIVIUS), Department of Physics of Condensed Matter, Optica Area, Faculty of Pharmacy, University of Seville, 41012 Seville, Spain; 2 Department of Ophthalmology, Clínica Novovisión, 30107 Murcia, Spain

**Keywords:** small aperture intraocular lens, IC-8 intraocular lens, extended-depth-of-focus lens, irregular cornea, cataract, refractive surgery, presbyopia

## Abstract

The aim of this paper is to evaluate the visual outcomes and patient satisfaction of small aperture IC-8 IOLs in cataract patients with or without prior ocular events. A systematic review of full-length original English studies reporting the visual results of small aperture IC-8 IOL implantation after cataract surgery in three databases, PubMed, Web of Science and Scopus, was performed according to the PRISMA statement. The Quality Assessment Tool for case series studies from the National Heart, Lung, and Blood Institute was used to analyze the quality of the studies selected. The search provided 543 articles, of which 22 were included in this systematic review. Significant improvements in uncorrected distance visual acuity (UDVA); uncorrected intermediate visual acuity (UIVA); uncorrected near visual acuity (UNVA); perception of photic phenomena; and patient satisfaction have been reported. Unilateral and bilateral small aperture IC-8 IOL implantation reduces photic phenomena and provides good vision for all distances with high patient satisfaction and minimal postoperative complications. Therefore, the implantation of this IOL may be recommended for patients with cataracts, corneal irregularities and ocular trauma with partial aniridia.

## 1. Introduction

Cataracts are one of the main causes of visual impairment globally [1]. Cataract patients are reported to have a higher anxiety and depression risk as a result of their dependence on others in their daily living activities [2,3,4]. In addition, in a recent systematic review and meta-analysis, the leading cause of blindness in 2015 was cataracts (12.6 million), and this number increased by 2020 (13.4 million) [5]. It is estimated that 20 million people are blind due to cataracts [6]. Thus, cataract surgery significantly benefits the visual function and patients’ quality of life [7,8,9,10].

Currently, increased life expectancy and changes in lifestyle have increased the patients’ visual demands [11]. Therefore, cataract patients tend to demand spectacle independence after surgery [12,13]. However, there are situations that influence cataract surgery success, such as previous corneal irregularities. These corneal irregularities are mainly caused by keratoconus, penetrating keratoplasty, refractive surgery and corneal scarring, inducing high-order aberrations (HOAs), which have an impact on patients’ visual acuity (VA) [14]. In addition, the most popular IOL power calculators do not consider the differences between the anterior and posterior refraction of the cornea, thus resulting in an erroneous IOL power calculation and postoperative refractive surprises. However, other new generation formulas include posterior cornea and lens thickness in IOL power calculation [15].

Monofocal IOLs provide the best possible distance vision, but they are not the desired option because these lenses do not meet the visual demand at all distances [16]. Although multifocal IOLs supply good functional vision, they provide a noncontinuous range of vision and reduce contrast sensitivity [17,18,19]. In addition, they are limited by photic phenomena such as glare and halos due to their diffractive optics [20,21]. Extended-depth-of-focus (EDOF) IOLs are a relatively novel technology for the treatment of presbyopia. EDOF IOL technology creates a single elongated focal point, providing a continuous range of vision with excellent distance vision, improved intermediate vision and functional near vision [11].

The small aperture IC-8 IOL (AcuFocus Inc., Irvine, CA, USA) is a newer EDOF IOL based on the KAMRA corneal inlay design (AcuFocus Inc., Irvine, CA, USA) [22]. The small aperture IC-8 IOL is a single piece hydrophobic acrylic posterior chamber IOL that combines pinhole technology through a central 3.23 mm black circular mask composed of polyvinylidene difluoride and carbon nanoparticles with a central 1.36 mm aperture. The opaque mask has over 3200 microperforations and does not influence patients’ field of vision [23]. This IOL has modified c haptics with an overall diameter of 12.5 mm and is available in +15.5 to +27.5 D range. Some published studies in cataract patients have suggested that small aperture IC-8 IOLs provide uncorrected good vision for all distances with a maintenance sensitivity of contrast [24,25,26,27,28,29,30]. Moreover, the design of this IOL provides an opportunity to enhance vision in patients with ocular trauma or corneal irregularities [31].

To our knowledge, there is no systematic review exploring all the literature available on the topic of small aperture IC-8 IOL. The purpose of this study was to systematically review case series of visual outcomes and satisfaction after small aperture IC-8 IOL implantation in patients with cataracts, corneal irregularities and ocular trauma in the available scientific literature.

## 2. Materials and Methods

This systematic review was carried out by searching the PubMed, Web of Science and Scopus databases on 15 July 2022. A Cochrane search had retrieved zero results. The study was performed according to the recommendation of the Preferred Reporting Items for Systematic Reviews and Meta-Analyses (PRISMA) [32,33]. An initial search was conducted, focused on obtaining case studies of small aperture IOLs in cataract surgery. The search strategy was *“(Small Aperture OR Pinhole OR IC-8) AND (Intraocular Lens OR IOL OR Cataract Surgery OR Lensectomy Surgery OR Refractive Surgery)”.* From them, a total of 543 articles were identified, which were evaluated and selected according to the inclusion and exclusion criteria. The inclusion criteria were: (1) small aperture IC-8 IOL in standard cataract, irregular cornea with or without prior surgery and ocular trauma. The exclusion criteria were as follows: (2) narrative reviews; (3) animal studies; (4) editorials or letters to the editor; (5) publications within XtraFocus Small Aperture EDOF IOL; (6) articles without findings or conclusions; (7) articles in nonindexed scientific journals.

The following data are summarized in tables: (1) authors and year of publication; (2) study design; (3) maximum follow-up period expressed in months; (4) number of patients; (5) number of eyes implanted; (6) sex; (7) refractive target in diopters; (8) past medical history, namely, previous surgeries; (10) preoperative mean refractive spherical equivalent (MRSE); (11) postoperative MRSE; (12) percentage of eyes within 20/32 or better (Jaeger 3) of uncorrected near visual acuity (UNVA); (13) percentage of eyes within 20/32 or better (Snellen) uncorrected intermediate visual acuity (UIVA); (14) percentage of eyes within 20/32 or better (Snellen) of uncorrected distance visual acuity (UDVA); (15) patient satisfaction rate, expressed in score points from 0 to 10; (16) postoperative complications after Small Aperture IOL.

To assess the risk of bias of the included studies, a summary table was elaborated (Table 1) based on the Quality Assessment Tool for Case Series Studies from the National Heart, Lung, and Blood Institute [34]. The questions included in the tool were as follows: (1) Is the study oriented to a clear question? (2) Were all the patient results taken into account? (3) Was the follow-up complete? (4) Were the same conditions used in surgical treatment? (5) Was the intervention clearly described? (6) Was the duration of follow-up adequate? (7) Were the results described correctly? This analysis did not result in the exclusion of any article. However, articles with a higher risk of bias had a lower weight for the data synthesis conclusions. The risk of bias was assessed by J.M., S.G. and A.B.-S. In case of disagreements, C.D.-H.-C. decided the tie-breaker.

## 3. Results

**Figure 1:**
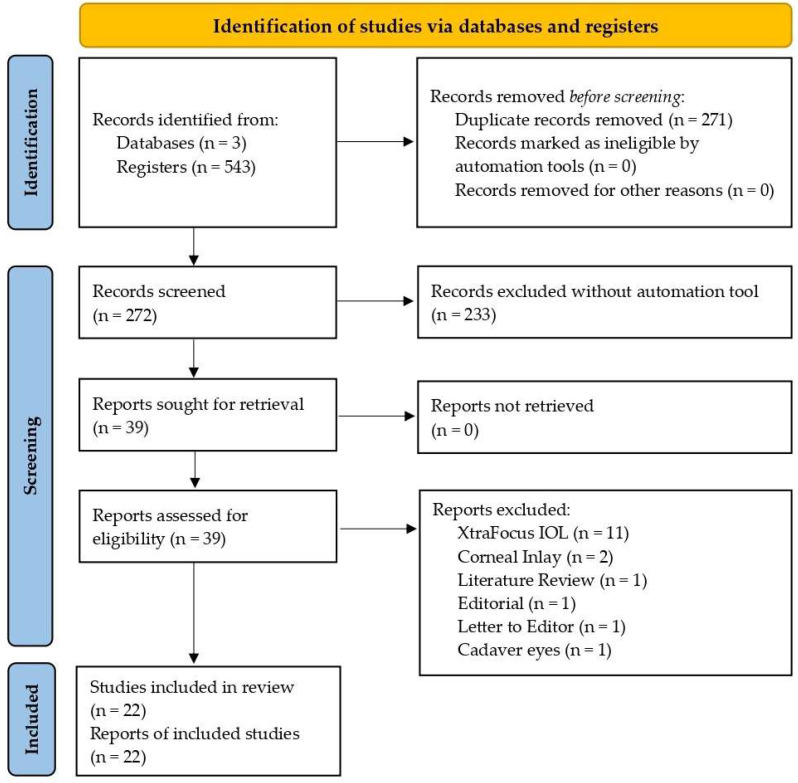
Flowchart study selection process according to the PRISMA statement.

The selection process of this systematic review is presented with a flow chart diagram in Figure 1. A total of twenty-two articles [23,24,25,26,27,28,29,30,35,36,37,38,39,40,41,42,43,44,45,46,47,48] published between 2015 and 2022 were included in this systematic review. All of them were cases reports or case series studies. The general inclusion criteria for all the studies were patients older than 18 years with substantial unilateral or bilateral cataracts, grade I to V in the Lens Opacities Classification System III, seeking spectacle independence and with a preexisting corneal regularity or irregularity. Previous corneal events including corneal trauma; ocular perforation; aniridia; keratoconus; LASIK; radial keratotomy (RK); penetrating keratoplasty (PK); or IntraCOR refractive surgery were among the included patients. Exclusion criteria included monophthalmic and microphthalmic patients; previous ocular surgery including chronic or recurrent uveitis; acute ocular disease or external/internal infection; diabetes mellitus with retinal changes; glaucoma or intraocular pressure equal to or higher than 24 mm Hg; pseudoexfoliation syndrome; pathological miosis or pupillary irregularity; and corneal endothelial dystrophy. Patient and surgery details of the selected articles are reported in Table 2.

This systematic review included 460 eyes from a total of 443 patients, and the maximum postoperative follow-up ranged from 1 to 23.6 months with a mean maximum follow-up of 6.16 months. Twelve [23,24,25,26,28,29,37,39,40,41,44,48] of the twenty-two included studies reported AcuFocus disclosure, including disclosure by clinical investigators, AcuFocus employees, medical advisors, consultants, physician advisors, research grants and personal fees. The mean age of the patients was 61.92 ± 11.62 years old and ranged from 17 to 73 years. The gender distribution within the studies that reported sex was 224 (55.58%) males and 179 (44.42%) females. The mean myopic target for the nondominant small aperture IC-8 IOL was −0.61 ± 0.44 diopters and ranged from 0.00 to −1.73 diopters.

The small aperture IOL outcomes are presented in Table 3. Concerning the previous ocular history of patients, there were 10 articles [23,24,25,26,27,28,29,30,40,41] that studied cataract surgery without prior surgery or ocular events and twelve articles [35,36,37,38,39,42,43,44,45,46,47,48] that studied small aperture IOL implantation with prior ocular events.

The preoperative mean refractive spherical equivalent was 0.28 ± 2.54 diopters and ranged from −3.18 to +5.43 diopters. At the last visit, the postoperative mean refractive spherical equivalent was −0.53 ± 1.18 diopters and ranged from −2.12 to +3.50 diopters. In the postoperative period, there was a notable improvement in UNVA and UIVA. At the last follow-up appointment, UNVA ranged between 0% and 100% of eyes with 20/32 or better, with a mean UNVA of 20/32 or better in 52.08% of eyes. UIVA ranged between 11.7% and 100% of eyes with 20/32 or better. The mean UIVA was 81.22%. UDVA ranged between 33.3% and 100% with 20/32 or better, with a mean UDVA of 91.09% of eyes with 20/32 or better. The exact distances in which UDVA, UIVA and UNVA were measured were not reported in the included articles. Furthermore, seven articles [24,25,26,27,28,29] offered information on patient satisfaction, and the overall score was between 7.28 and 8.99 out of 10. The mean satisfaction score was 8.23 ± 0.65 points. Regarding complications, hyphema, elevated intraocular pressure, cystoid macular edema, IOL exchange and posterior capsular opacification (PCO) were reported.

Finally, the included studies were grouped into three levels based on the risk of bias assessment tool. The groups were low evidence (affirmative answers = 0 to 2); medium evidence (affirmative answers = 3 to 5); and high evidence (affirmative answers = 6 to 7). No studies reported a low evidence level. Schultz and Dick [35]; Agarwal and Thornell [36]; Barnett et al. [38]; Son et al. [40]; Srinivasan et al. [23]; Agarwal and Thornell [42]; Son et al. [44]; Hartmann et al. [45]; Langer et al. [46]; Northey et al. [47]; and Baur et al. [48] reported medium evidence levels. Grabner et al. [24]; Dick et al. [25]; Ang [37]; Barnett et al. [38]; Dick et al. [26]; Ang [39]; Hooshmand et al. [27]; Ang [41]; Ang et al. [28]; Schojai et al. [29]; Shajari et al. [43]; and Yang et al. [30] achieved high evidence levels.

## 4. Discussion

### 4.1. Visual Outcomes and Satisfaction

Nineteen of the studies [24,25,26,27,28,29,30,35,36,38,40,41,42,43,44,45,46,47,48] included in this systematic review have evaluated improvements in vision and the perception of photic phenomena after implantation of the IC-8 small aperture intraocular lens.

Regarding visual outcomes, eight studies [24,25,26,27,29,30,40,41] assessed UDVA, UIVA and UNVA in cataract patients without prior ocular events. Dick et al. [25] included 105 patients who received small aperture IC-8 implantation in the nondominant eye and a monofocal IOL in the fellow eye. They reported that 99%, 95% and 79% of eyes achieved a UDVA, UIVA and UNVA of 20/32 or better, respectively. In a similar study conducted by Hooshmand et al. [27], 126 patients who received small aperture IC-8 implantation in the nondominant eye and a monofocal or multifocal IOL in the fellow eye were included. They reported that 99%, 95% and 79% of eyes reached a UDVA, UIVA and UNVA of 20/32 or better, respectively. Similar results were obtained by Grabner et al. [24], Dick et al. [26], Son et al. [40], Ang [41] and Schojai et al. [29]. However, Yang et al. [30] reported that only 58% of eyes attained a UDVA of 20/32 or greater, which may be because the mean MRSE after small aperture IC-8 implantation was −0,84 D.

Ten studies [35,36,38,42,43,44,45,46,47,48] also evaluated UDVA, UIVA and UNVA in patients with previous ocular events. Shajari et al. [43] assessed 17 patients who received unilateral small aperture IC-8 IOL implantation in eyes with keratoconus, RK or PK. They reported that 88.2% and 11.7% of eyes achieved a UDVA and UIVA of 20/32 or higher, respectively. In addition, no eyes exceeded a UNVA of 20/32. Langer et al. [46] also reported that no eye exceeded a UNVA of 20/32 after unilateral small aperture IC-8 IOL implantation in 17 patients with keratoconus, RK or PK. However, it is important to mention that postoperative UIVA and UNVA improved significantly in both studies. Similar results were reported by Agarwal and Thornell [36,42], Barnett et al. [38], Hartmann et al. [45], Northey et al. [47] and Baur et al. [48], who included patients with LASIK, RK, keratoconus and IntraCOR refractive surgery, respectively. Schultz and Dick [35] and Son et al. [44] also reported that 100% and 33.3% of eyes achieved a UDVA of 20/32 or greater after small aperture IC-8 IOL implantation in four eyes with partial aniridia due to ocular trauma. The preoperative UDVA was 20/100 and 20/160 in both studies, respectively.

Regarding photic phenomena, eight studies [24,25,26,27,28,29,40,41] reported symptoms of halos and glare in cataract patients without any previous ocular events. Grabner et al. [24], Dick et al. [25,26], Hooshmand et al. [27] and Ang [41] further assessed symptoms of halos and glare on a scale from 0 to 10, where 0 indicated no photic phenomena and 10 indicated high photic phenomena. They reported that the overall symptoms of halos and glare were low. In addition, glare and halo scores are lower in EDOF IOLs than in multifocal IOLs [49,50]. However, Hooshmand et al. [27] obtained the highest symptoms of halos and glare, with scores of 5.4 and 5.6, respectively. This may be because seven patients developed an incipient PCO, which is related to more symptoms of glare and light sensitivity [51,52].

Five studies [35,36,42,44,48] also evaluated the perception of photic phenomena in patients with prior ocular events. Agarwal and Thornell [36,42] reported no symptoms of glare and halos after small aperture IC-8 IOL implantation in patients with LASIK and RK. However, Schultz and Dick [35] and Son et al. [44] reported photic phenomena in three patients with partial aniridia due to ocular trauma. Baur et al. [48] also reported similar results in one patient who received IntraCOR refractive surgery. Although these studies reported symptoms of halos and glare after small aperture IC-8 IOL implantation in patients with prior ocular events, it is important to mention that postoperative photic phenomena were lower than preoperative.

Regarding patient satisfaction, Grabner et al. [24], Dick et al. [25,26], Hooshmand et al. [27], Ang [41], Ang et al. [28], and Schojai et al. [29] assessed this variable with the satisfaction questionnaire score, where a score of 1 indicated very dissatisfied and 10 very satisfied. All these studies reported a high satisfaction questionnaire score after small aperture IC-8 IOL implantation. However, Grabner et al. [24] reported the lowest satisfaction score of 7.28. This may be because Grabner et al. [24] only assessed patient satisfaction at near vision, whereas Dick et al. [25,26], Ang [41], and Schojai et al. [29] reported overall patient satisfaction. In addition, EDOF IOLs provide functional near vision, but in some cases, patients may need a spherical addition to optimize it. Therefore, it is common for patient satisfaction in near vision to be lower than in intermediate and distance vision.

The improvements in visual acuity, photic phenomena, and satisfaction after small aperture IC-8 implantation are due to its design. Pinhole technology artificially reduces the patient’s pupil size; therefore, a decrease in HAOs improves vision and photic phenomena, increasing patients’ visual satisfaction.

### 4.2. Complications

Advances in cataract surgery have significantly reduced intraoperative and postoperative complications. Schultz and Dick [35], Barnett et al. [38], Srinivasan et al. [23], Schojai et al. [29], Son et al. [44], and Hartmann et al. [45] reported no complications after small aperture IC-8 IOL implantation. Ang et al. [28] and Shajari et al. [43] reported four posterior capsular opacifications (PCOs) that were treated with YAG capsulotomy without any complications. However, Hooshmand et al. [27] reported seven IOL exchanges due to incipient PCO that could not be solved with YAG capsulotomy. Grabner et al. [24] reported one case of hyphemia, and Dick et al. [25] reported two cases of elevated intraocular pressure, which was due to postoperative corticosteroid administration, and one case of cystoid macular edema. All the complications reported by the articles included in this systematic review were the most frequent after cataract surgery [53,54].

### 4.3. Unilateral vs. Bilateral

All studies included in this systematic review evaluated patients with unilateral implantation of small aperture IC-8 IOLs in the nondominant eye. Ang [37], Dick et al. [26], and Ang et al. [28] further assessed visual outcomes and patient satisfaction after unilateral and bilateral implantation of small aperture IC-8 IOL.

Ang [37] reported the overall visual improvements and not by group. Dick et al. [26] included 11 and six patients with unilateral and bilateral implantation, respectively. He reported that 100% of patients achieved a UDVA of 20/32 or better in both groups. Concerning UIVA, he reported that 100% and 82% of the patients obtained a visual acuity of 20/32 or better in the unilateral and bilateral groups, respectively. However, 50% of bilateral implantation patients reached a visual acuity of 20/16, while no unilateral implantation patients obtained this visual acuity. Regarding UNVA, he reported that 82% and 100% of the patients achieved a visual acuity of 20/32 or better in the unilateral and bilateral groups, respectively. Similar results were reported by Ang et al. [28], who included 10 patients with unilateral and bilateral implantation. They reported that 100% of all patients obtained a visual acuity of 20/32 or better for all distances. In addition, they also reported that UIVA and UNVA were 0.5 to 1 line greater in bilateral patients. These studies suggest that bilateral implantation of small aperture IC-8 IOL provides better intermediate and near vision than unilateral implantation, which may be due to a greater extension of the depth of focus achieved after bilateral implantation.

Regarding patient satisfaction, Dick et al. [26] reported that patients with bilateral implantation had a higher perception of photic phenomena. Therefore, satisfaction was lower than in patients with unilateral implantation. However, Ang et al. [28] found no significant differences in patient satisfaction in either group. Different enrollment criteria between studies may explain this difference. In the study by Dick et al. [26], a small aperture IC-8 IOL was implanted simultaneously in the bilateral group. However, Ang et al. [28] adjusted the refractive target of a second IOL implantation, reducing possible visual discomfort generated after the first IOL implantation. In addition, Dick et al. [26] also reported that the preoperative pupil diameter was larger in the bilateral group. Preoperative mesopic pupil diameters of 5.6 mm or more induce higher photic phenomena and, thus, lower patient satisfaction after small aperture IC-8 IOL implantation [25].

### 4.4. Strengths and Limitations

This is the first systematic review that describes visual outcomes and patient satisfaction after small aperture IC-8 IOL implantation in patients with cataracts, corneal irregularities and ocular trauma. The main limitation of our review is that all the studies included are case reports or series of cases, many of them with a small sample size and short-term follow-up periods. No randomized clinical trials were included, as there are none in the available literature.

## 5. Conclusions

Unilateral small aperture IC-8 IOL implantation provides great distance and intermediate vision with functional near vision, less photic phenomena, and thus a high overall satisfaction in cataract patients, corneal irregularities and ocular trauma with partial aniridia. Bilateral small aperture IC-8 IOL implantation seems to improve intermediate and near vision. Postoperative complications are similar to other IOLs.

## Figures and Tables

**Table 1:** Quality assessment of articles.

Author (Date)	Q1	Q2	Q3	Q4	Q5	Q6	Q7
Grabner et al. [24] (2015)	Yes	Yes	Yes	Yes	Yes	Yes	Yes
Schultz and Dick [35] (2016)	Yes	Yes	Yes	NA	Yes	No	No
Dick et al. [25] (2017)	Yes	Yes	Yes	Yes	Yes	No	Yes
Agarwal and Thornell [36] (2018)	Yes	Yes	Yes	No	Yes	No	No
Ang [37] (2018)	Yes	Yes	Yes	Yes	Yes	No	Yes
Barnett et al. [38] (2018)	Yes	Yes	Yes	NA	Yes	No	No
Dick et al. [26] (2018)	Yes	Yes	Yes	Yes	Yes	No	Yes
Ang [39] (2019)	No	Yes	Yes	Yes	Yes	Yes	Yes
Hooshmand et al. [27] (2019)	Yes	Yes	Yes	Yes	Yes	No	Yes
Son et al. [40] (2019)	Yes	Yes	Yes	Yes	Yes	No	No
Srinivasan et al. [23] (2019)	No	Yes	Yes	Yes	Yes	No	Yes
Ang [41] (2020)	Yes	Yes	Yes	Yes	Yes	Yes	Yes
Ang et al. [28] (2020)	Yes	Yes	Yes	Yes	Yes	No	Yes
Agarwal and Thornell [42] (2020)	Yes	Yes	Yes	No	Yes	No	No
Schojai et al. [29] (2020)	Yes	Yes	Yes	Yes	Yes	No	Yes
Shajari et al. [43] (2020)	Yes	Yes	Yes	Yes	Yes	No	Yes
Son et al. [44] (2020)	Yes	Yes	Yes	No	Yes	Yes	No
Hartmann et al. [45] (2021)	No	Yes	Yes	No	Yes	No	No
Langer et al. [46] (2021)	Yes	Yes	Yes	No	Yes	No	No
Northey et al. [47] (2021)	Yes	Yes	Yes	No	Yes	No	No
Baur et al. [48] (2022)	Yes	Yes	Yes	NA	Yes	No	Yes
Yang et al. [30] (2022)	Yes	Yes	Yes	Yes	Yes	No	Yes

NA: not applicable; Q = question. (Q1) Is the study oriented to a clear question? (Q2) Were all the patients results taken into account? (Q3) Was the follow-up complete? (Q4) Were the same conditions used in surgical treatment? (Q5) Was the intervention clearly described? (Q6) Was the duration of follow-up adequate? (Q7) Were the results described correctly?

**Table 2:** Study characteristics.

Author (Date)	Design	AcuFocus Disclosure	Follow-Up (Months)	Patients	Eyes	IOL Side	Age (Years)	Sex (F/M)	Refractive Target (D)
Grabner et al. [24] (2015)	CS	CI and Employee	12	12	12	ML	60.5	9/3	−0.75
Schultz and Dick [35] (2016)	CR	None	6	1	1	ML	17.0	0/1	0.00
Dick et al. [25] (2017)	CS	CI, MA and Employee	6	105	105	ML	67.5	60/45	−0.50
Agarwal and Thornell [36] (2018)	CS	None	6	3	3	ML	67.6	NR	−0.25
Ang [37] (2018)	CS	CI and MA	NR	10	11	ML and BL	65.1	6/4	NR
Barnett et al. [38] (2018)	CR	None	1	1	1	ML	73.0	0/1	−0.50
Dick et al. [26] (2018)	CS	Consultant and PA	6	17	23	ML and BL	NR	NR	−0.50
Ang [39] (2019)	CS	CI and MA	23.6	12	12	ML	62.4	5/7	NR
Hooshmand et al. [27] (2019)	CS	None	6.76	126	126	ML	68.0	64/62	−0.75
Son et al. [40] (2019)	CS	Research Grants	5	13	13	ML	68.5	9/4	−0.50
Srinivasan et al. [23] (2019)	CS	MA	NR	15	15	ML	NR	NR	NR
Ang [41] (2020)	CS	Research Grants	12	20	30	ML and BL	62.6	13/7	−0.50
Ang et al. [28] (2020)	CS	CI and Research Grants	3	30	30	ML	60.7	20/10	−0,75
Agarwal and Thornell [42] (2020)	CS	None	6	4	4	ML	69.7	NR	−0.75
Schojai et al. [29] (2020)	CS	MA	3	18	18	ML	69.0	12/6	−0.75
Shajari et al. [43] (2020)	CS	None	3	17	17	ML	54.0	9/8	0.00
Son et al. [44] (2020)	CS	Research Grants	12	3	3	ML	65.6	0/3	−1.50
Hartmann et al. [45] (2021)	CS	None	6	2	2	ML	62.5	NR	−0.75
Langer et al. [46] (2021)	CS	None	3	17	17	ML	54.0	9/8	0.00
Northey et al. [47] (2021)	CS	None	3	4	4	ML	63.0	2/2	−1.73
Baur et al. [48] (2022)	CR	Research Grant and PF	3	1	1	ML	66.0	0/1	−0.75
Yang et al. [30] (2022)	CS	None	3	12	12	ML	61.7	6/6	−0.50

CS: case series; CI: clinical investigators; F/M = female/male; IOL: intraocular lens; ML: monolateral; BL: bilateral; CR: case report; MA: medical advisor; NR = not reported; PA: physician advisor; PF: personal fees.

**Table 3:** Evaluation of the visual results after the implantation of Small Aperture Intraocular Lens.

Author (Date)	Previous History	Pre MRSE (D)	Post MSRE (D)	UNVA *	UIVA *	UDVA *	Photic Phenomena	Satisfaction **	Complications (*n*)
Grabner et al. [24] (2015)	Cataract	+0.95	−0.10	92	100	100	Glare and Halo	7.28	Hyphema (1)
Schultz and Dick [35] (2016)	Cornea Trauma	NR	NR	100	NR	100	Glare	NR	None
Dick et al. [25] (2017)	Cataract	+0.30	−0.42	79	95	99	Glare and Halo	8.6	↑ IOP (2) CME (1)
Agarwal and Thornell [36] (2018)	LASIK	NR	−0.69	0	100	100	None	NR	NR
Ang [37] (2018)	RC	−0.57	NR	NR	NR	NR	NR	NR	NR
Barnett et al. [38] (2018)	RK	+5.43	+3.50	NR	NR	100	NR	NR	None
Dick et al. [26] (2018)	Cataract	NR	NR	82	100	100	Glare and Halo	7.5	NR
Ang [39] (2019)	RC	−0.61	NR	NR	NR	NR	NR	NR	NR
Hooshmand et al. [27] (2019)	Cataract	+0.60	NR	76.2	83.3	98	Glare and Halo	8.6	IOL Exchange (7)
Son et al. [40] (2019)	Cataract	NR	−0.43	NR	NR	100	Glare and Halo	NR	NR
Srinivasan et al. [23] (2019)	Cataract	NR	NR	NR	NR	NR	NR	NR	None
Ang [41] (2020)	Cataract	+0.87	−0.50	100	100	100	Glare	8.2	NR
Ang et al. [28] (2020)	Cataract	NR	−0.17	NR	NR	NR	Glare and Halo	8.78	PCO (2)
Agarwal and Thornell [42] (2020)	RK	+0.08	−1.08	25	100	100	None	NR	NR
Schojai et al. [29] (2020)	Cataract	NR	−0.53	85	100	100	Glare and Halo	8.99	None
Shajari et al. [43] (2020)	KC/RK/PK	NR	−1.22	0	11.7	88.2	NR	NR	PCO (2)
Son et al. [44] (2020)	PK/Aniridia/OP	−2.91	−0.81	NR	NR	33.3	Halo	NR	None
Hartmann et al. [45] (2021)	RK	+4.00	−1.25	NR	NR	100	NR	NR	None
Langer et al. [46] (2021)	KC/RK/PK	NR	−1.22	0	23.5	88.2	NR	NR	NR
Northey et al. [47] (2021)	KC	−3.18	−2.12	0	NR	75	NR	NR	NR
Baur et al. [48] (2022)	IntraCOR	+1.75	−0.62	0	NR	100	Halo	NR	NR
Yang et al. [30] (2022)	Cataract	−2.99	−0.84	90	80	58	NR	NR	NR

MRSE: mean refractive spherical error; UNVA: uncorrected near visual acuity; UIVA: uncorrected intermediate visual acuity; UDVA: uncorrected distance visual acuity; NR: not reported; IOP: intraocular pressure; CME: cystoid macular edema; PCO: posterior capsular opacification; LASIK: laser assisted in situ keratomileusis; RK: radial keratotomy; RC: refractive candidate; IOL: intraocular lens; KC: keratoconus; PK: penetrating keratoplasty; OP: ocular perforation. * Percentage of eyes with 20/32 (Snellen for UDVA and UIVA and Jaeger 3 for UNVA) or better. ** Score point in a 0 to 10 scale.

## Data Availability

Not applicable.
